# An inhibitory compound produced by a soil isolate of *Rhodococcus* has strong activity against the veterinary pathogen *R*. *equi*

**DOI:** 10.1371/journal.pone.0209275

**Published:** 2018-12-28

**Authors:** Amber L. Ward, Pushpavathi Reddyvari, Ralitsa Borisova, Abbas G. Shilabin, Bert C. Lampson

**Affiliations:** 1 Department of Health Sciences, East Tennessee State University, Johnson City, TN, United States of America; 2 Department of Chemistry, East Tennessee State University, Johnson City, TN, United States of America; Universidad Nacional Autonoma de Mexico Facultad de Quimica, MEXICO

## Abstract

Complete genome sequencing of dozens of strains of the soil bacterium *Rhodococcus* has revealed the presence of many cryptic biosynthetic gene clusters, presumably dedicated to the production of small molecules. This has sparked a renewed interest in this underexplored member of the *Actinobacteria* as a potential source of new bioactive compounds. Reported here is the discovery of a potent inhibitory molecule produced by a newly isolated strain of *Rhodococcus*, strain MTM3W5.2. This small inhibitory molecule shows strong activity against all *Rhodococcus* species tested, including the veterinary pathogen *R*. *equi*, and some closely related genera. It is not active against other Gram positive or Gram negative bacteria. A screen of random transposon mutants identified a gene required to produce this inhibitory compound. This gene is a large multi-domain, type I polyketide synthase that is part of a very large multi-gene biosynthetic gene cluster in the chromosome of strain MTM3W5.2. The high resolution mass spectrum of a major chromatogram peak from a broth culture extract of MTM3W5.2 shows the presence of a compound at *m/z* 911.5490 atomic mass units. This compound is not detected in the culture extracts from a non-producing mutant strain of MTM3W5.2. A large gene cluster containing at least 14 different type I polyketide synthase genes is proposed to be required to synthesize this antibiotic-like compound.

## Introduction

For decades there has been a decline in the discovery of new antibiotics that have a novel structure and mode of action and thus more likely to be effective against bacteria resistant to older line antibiotics. For example, no new antibiotic “scaffold” was discovered between 1962 and 2000 [1]. However, recent DNA sequencing of bacterial genomes has revealed many unknown genes apparently devoted to production of secondary metabolites (often small organic molecules) that do not appear to be expressed when bacteria are cultured in the lab. Thus, perhaps ninety percent of the potential small molecules that can be produced by bacteria remain unknown [2].

As noted in a recent study, the bacterial genus *Rhodococcus* may represent a mostly under-explored source of new small molecules based on the large number of cryptic secondary metabolite, biosynthetic gene clusters (BGC) found in most newly sequenced genomes of different rhodococci [3]. The rhodococci are mostly soil bacteria that are Gram positive but also contain mycolic acids in their cell wall. They grow initially as long branching filaments that then fragment into short rods or cocci [4]. They are members of the *Actinomycetales* and are related to prolific small molecule producers like *Streptomyces* [5, 6]. *Rhodococcus equi* (see reference [S8] for alternate names for this bacterium) is an important veterinary pathogen that causes a severe bronchopneumonia in foals, often less than seven months of age. *R*. *equi* can also cause opportunistic infections in humans with a suppressed or weakened immune defense [7].

Although *Rhodococcus* species are well known for their remarkably diverse catabolic abilities [8], not much attention has been directed at the potential ability to produce bioactive small molecules. The siderophore compound heterobactin has been described [9], carotenoid pigments have been observed [10], and, prior to genome sequencing, three antibiotic type molecules have been reported to be produced by *Rhodococcus* strains. These include the antifungal rhodopeptins [11], the lariatins, so named because these lasso peptides contain an eight amino acid loop structure at their amino terminus [12], and finally the aurachins, which are very similar to the antibiotic aurachin C from the myxobacterium *Stigmatella aurantiaca* [13, 14]. In addition to these characterized molecules, a study by Kitagawa and Tamura [15] looked at about 80 different strains of *Rhodococcus* for antibacterial properties. They concluded that at least three different inhibitory compounds were produced from 15 different strains in their collection (14 of these positive strains were identified as *R*. *erythropolis*).

Many recently sequenced genomes of different *Rhodococcus* species appear to contain multiple gene clusters likely to be involved in the production of small molecules. These include polyketide synthases (PKS) and, especially common, are non-ribosomal peptide synthetases (NRPS)[3]. These types of enzymes are well known to be required to synthesize polyketide type molecules as well as small bioactive peptides [16]. Reported here is the discovery of a new strain of *Rhodococcus* that produces a potent inhibitory molecule with strong activity against the important veterinary pathogen *R*. *equi*. The inhibitory molecule has a narrow spectrum of activity with most species of *Rhodococcus* and members of closely related genera being sensitive to this molecule, while other Gram positive and Gram negative bacteria are not affected. The molecular mass of this compound was determined by LC-MS analysis and the genome sequence of the producer strain MTM3W5.2 revealed the BGC responsible for the production of this inhibitory molecule.

## Results

### The producer strain MTM3W5.2

**Fig 1:**
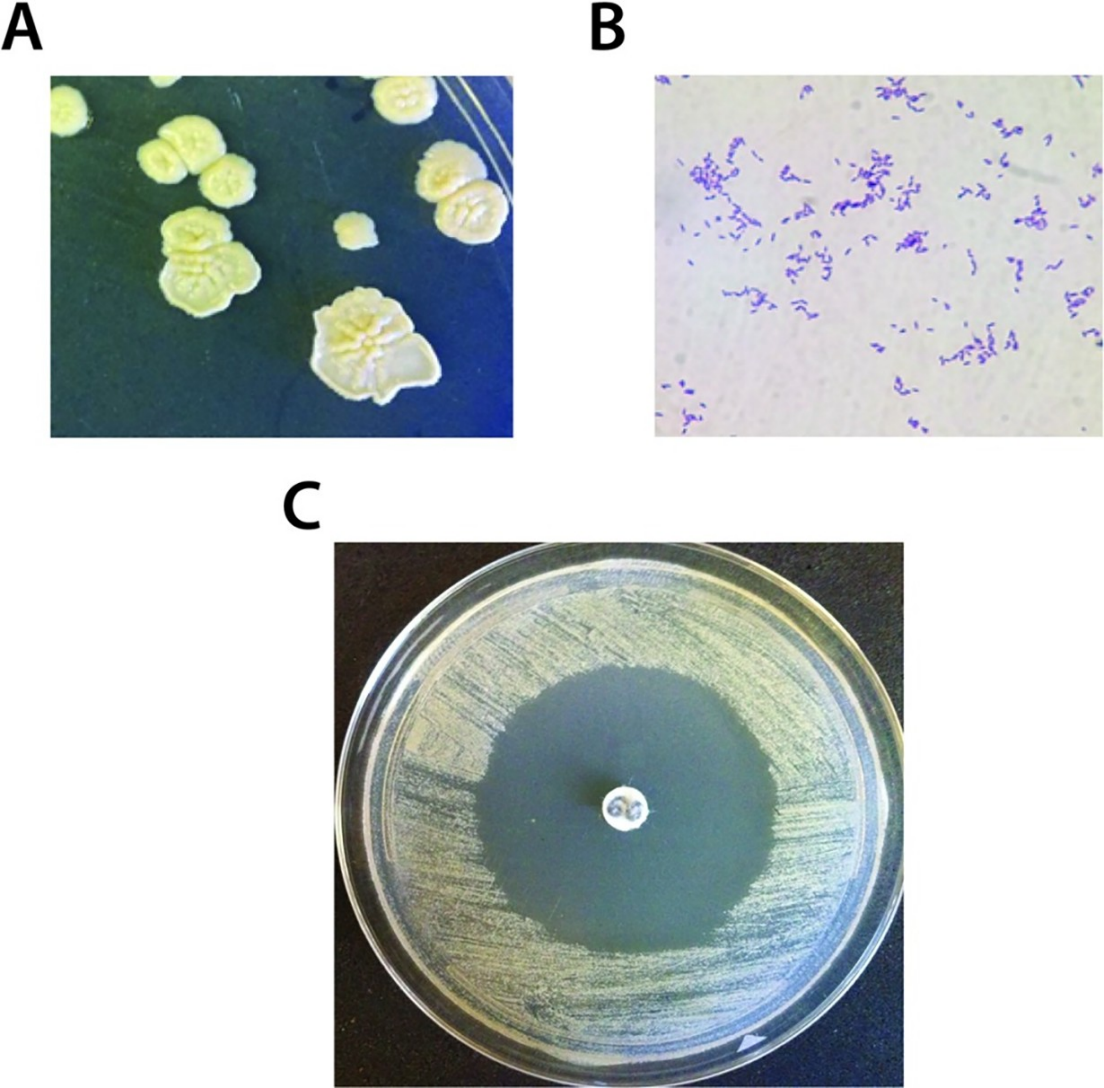
The producer strain *Rhodococcus* sp. MTM3W5.2. A. Colonies of *Rhodococcus* sp. MTM3W5.2, after three weeks of incubation, appear as large flat colonies with a slight yellow color. B. Broth cultures appear as a mix of lone and short Gram positive rods upon staining. C. Culture extracts, from strain MTM3W5.2 in a disk diffusion assay on the sensitive indicator strain *R*. *erythropolis* IGTS8, produce a large zone of no growth.

Ninety different strains of *Rhodococcus* were isolated and identified from different types of soil samples, mostly for the East Tennessee region (S1 Table). Despite the reported genomic potential to produce small bioactive molecules [3], only 3 out of 90 culture extracts prepared from this *Rhodococcus* collection showed any form of antibacterial activity (S1 Table). However, from a disk diffusion screen of agar plate culture extracts, a strain designated MTM3W5.2 revealed a strong inhibitory molecule. MTM3W5.2 was cultured from a lawn (surface) soil located in Morristown, Tennessee by the heat treatment method (see supplemental methods, S1 File). After three weeks at 22°C, it grows as large flat colonies on rich medium (RM) agar plates with a slight cream-yellow color (Fig 1A). Cells appear as a mix of long and short Gram positive rods upon staining (Fig 1B). DNA sequence analysis of the 16S ribosomal RNA gene indicates that strain MTM3W5.2 is most similar to the 16S rRNA sequence from *R*. *maanshanensis* strain M712 [17] (see also the genome below).

### The inhibitory molecule

After growth for two weeks on RM agar plates, MTM3W5.2 cells were washed off the plate and the agar medium was extracted with ethyl acetate. A potent inhibitory compound was detected from these extracts with strong activity against the sensitive indicator strain *R*. *erythropolis* strain IGTS8 (Fig 1C). This compound was not reliably detected from shaking broth cultures but is produced in stagnant broths of RM medium. Interestingly, the inhibitory compound is not produced at temperatures above about 22°C. However, RM plates grown at 20^o^, 15^o^, 10^o^, and 4°C all produced detectable levels of the inhibitory molecule.

A partially purified, HPLC fraction of the inhibitory compound was used to test its activity against a battery of other bacteria using a disk diffusion assay (Table 1). The inhibitory compound is active against all species and strains of *Rhodococcus* tested. The important veterinary pathogen *R*. *equi* was particularly sensitive to this molecule with a zone of inhibited growth of 38 millimeters (Table 1). Members of other genera closely related to *Rhodococcus* such as *Gordonia*, were also sensitive to the compound. It is noted however, that not all bacteria that produce mycolic acids (like *Mycobacterium smegmatis*) are sensitive to this compound and not all bacteria that do not contain mycolic acids are resistant (note the sensitivity of *Microbacterium* and *Agromyces*). In addition, the inhibitory molecule had no activity against any of the Gram negative bacteria tested. Also, two fungal organisms, *Aspergillus niger* and *Candida albicans* were not affected by this compound. As might be expected, the producer strain of *Rhodococcus*, MTM3W5.2, appears to be resistant to the inhibitor molecule it produces.

**Table 1:** Bacterial strain sensitivity to the inhibitory compound from strain MTM3W5.2.

Organism/strain	Taxon (Gram reaction)	Sensitive (zone size)	Reference^a^
*Rhodococcus erythropolis* IGTS8	Coryneform (+)	+ (40mm)	[9]
*Rhodococcus erythropolis* DP-45	“(+)	+ (17mm)	[34]
*Rhodococcus jostii* RHA1	“(+)	+ (35mm)	[35]
*Rhodococcus rhodochrous* ATCC 33279	“(+)	+ (25mm)	ATCC
*Rhodococcus ruber* 1979	“(+)	+ (26mm)	[36]
*Rhodococcus* sp. MTM3W5.2	“(+)	- (0mm)	This study
*Rhodococcus equi* ATCC 33701+	“(+)	+ (38mm)	ATCC
*Rhodococcus equi* MI 81–1267	“(+)	+ (35mm)	UT-COVM
*Rhodococcus equi* MI 98–3317	“(+)	+ (35mm)	UT-COVM
*Rhodococcus equi* MI 10–4887	“(+)	+ (35mm)	UT-COVM
*Rhodococcus equi* MI 17–43	“(+)	+ (36mm)	UT-COVM
*Rhodococcus equi* AAVLD 2001–4	“(+)	+ (34mm)	UT-COVM
*Rhodococcus equi* AAVLD 2005–2	“(+)	+ (32mm)	UT-COVM
*Gordonia* sp. BDHXW1B	“(+)	+ (27mm)	This study
*Corynebacterium diphtheriae* ATCC 11913	“(+)	+ (25mm)	ATCC
*Corynebacterium xerosis* ATCC 373	“(+)	+ (36mm)	ATCC
*Mycobacterium smegmatis* ATCC 700084	“(+)	- (0 mm)	ATCC
*Aeromicrobium* sp. SCTEC3	Nocardiodaceae (+)	- (0mm)	This study
*Microbacterium* sp. MTM3Y7	Micrococcales (+)	+ (30mm)	This study
*Agromyces* sp. BEM3Y1	“(+)	+ (20 mm)	This study
*Arthrobacter* sp. MTM3W2	“(+)	- (0 mm)	This study
*Cellulomonas* sp. NPDEW2	“(+)	- (0 mm)	This study
*Micrococcus luteus* ATCC 4698	“(+)	- (0 mm)	ATCC
*Bacillus subtilis* ATCC 6051	Firmicutes (+)	- (0 mm)	ATCC
*Staphylococcus aureus* ATCC 25923	“(+)	- (0 mm)	ATCC
*Staphylococcus saprophyticus* ATCC 15301	“(+)	- (0 mm)	ATCC
*Alcaligenes faecalis* ATCC 8750	Proteobacteria (-)	- (0 mm)	ATCC
*Pseudomonas aeruginosa* ATCC 10145	“(-)	- (0 mm)	ATCC
*Enterobacter aerogenes* ATCC 13048	Enterics (-)	- (0 mm)	ATCC
*Escherichia coli* ATCC 25922	“(-)	- (0 mm)	ATCC
*Klebsiella pneumoniae* ATCC 13883	“(-)	- (0 mm)	ATCC
*Pseudomonas aeruginosa* ATCC 10145	“(-)	- (0 mm)	ATCC
*Proteus vulgaris* ATCC 13315	“(-)	- (0 mm)	ATCC
*Salmonella arizonae* ATCC 13314	“(-)	- (0 mm)	ATCC
*Serratia marcescens* ATCC 13880	“(-)	- (0 mm)	ATCC
*Shigella sonnei* ATCC 29930	“(-)	- (0 mm)	ATCC
*Aspergillus niger*	Fungus	- (0 mm)	This study
*Candida albicans* ATCC 10213	Fungus	- (0 mm)	ATCC

^a^, ATCC, American type culture collection; UT-COVM, University of Tennessee College of Veterinary Medicine.

**Fig 2:**
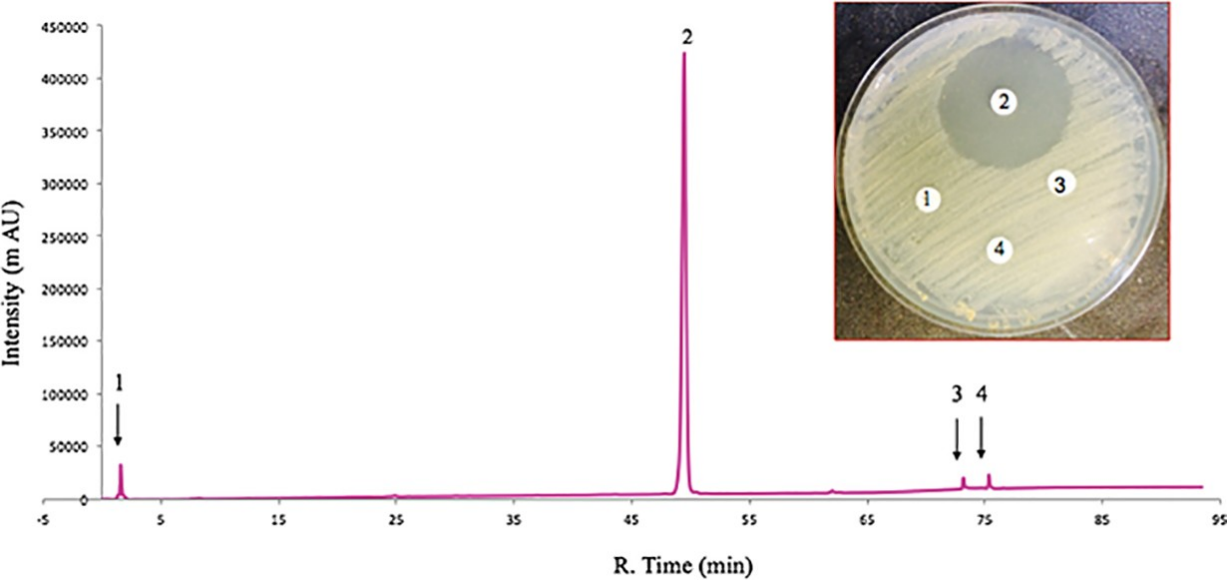
Purification of the inhibitory compound. The HPLC chromatogram shows that the pure compound elutes as a single large peak (peak 2) with a retention time of 48.0 minutes. Peak 2 is the only column fraction that shows antibacterial activity in a disk diffusion assay (picture inset).

**Fig 3:**
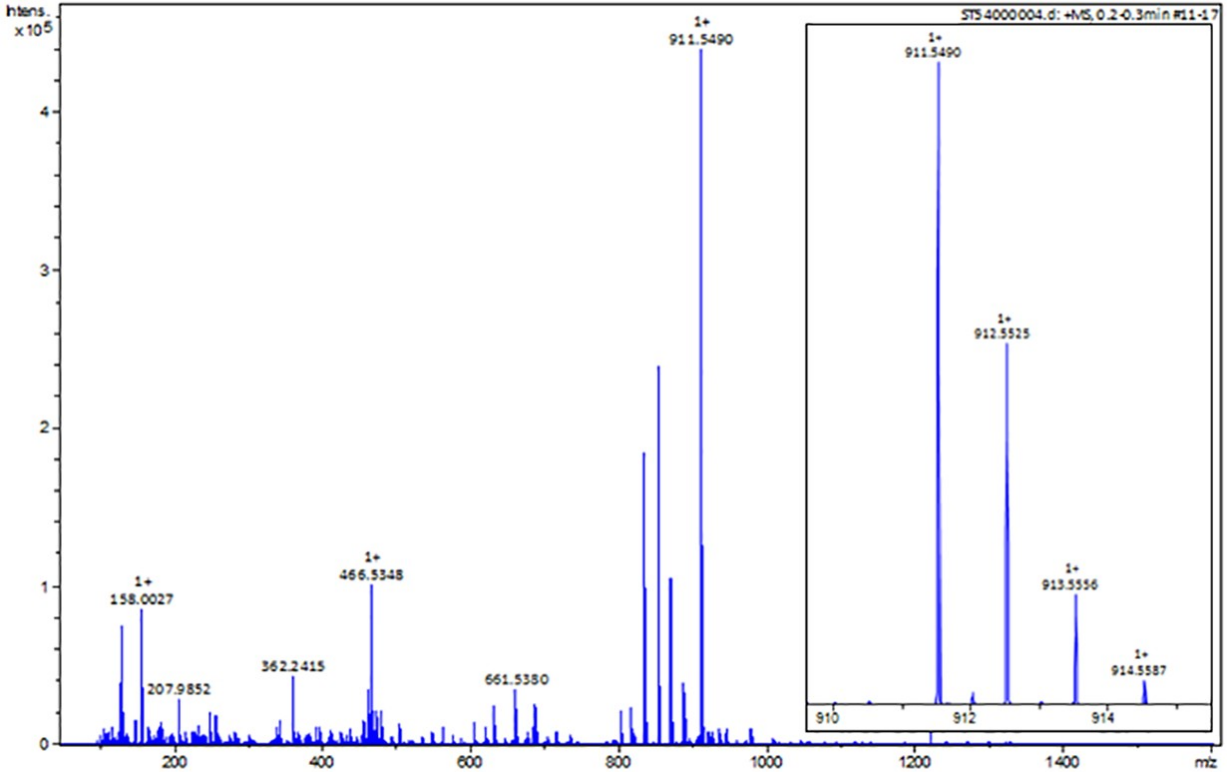
High-resolution mass spectra of the inhibitory compound. A full scan mass spectrum is shown for the pure inhibitory compound (peak 2, Fig 2) (large panel). A major ion signal appears with a mass of *m/z* 911.5490. A zoomed isotope cluster spectrum is shown for the major molecular ion peak (small panel). The exact mass of the inhibitory compound, derived from the ESI-Time of Flight Mass Spectrometer, is *m/z* 911.5490 [M + H]^+^.

The ability to produce this compound in stagnant broth cultures of MTM3W5.2 allowed for large-scale production and purification of the inhibitory molecule. The bacteria grow primarily as a biofilm on the surface of these large stagnant broths (500 mls of broth in a 2.8L flask). After extensive purification, the pure compound elutes as a single large peak from the HPLC column with a retention time of 48.9 minutes (Fig 2, peak 2). This is the only fraction showing inhibitory activity (Fig 2, picture inset). The pure compound (peak 2, Fig 2) was also analyzed by high resolution mass spectrometry (Fig 3) and determined to have a mass of *m/z* 911.5490 [M + H]. Based on this precise molecular mass, the molecular composition of the inhibitory compound is postulated to be C_52_H_78_O_13_.

### The non-producing mutants

**Fig 4:**
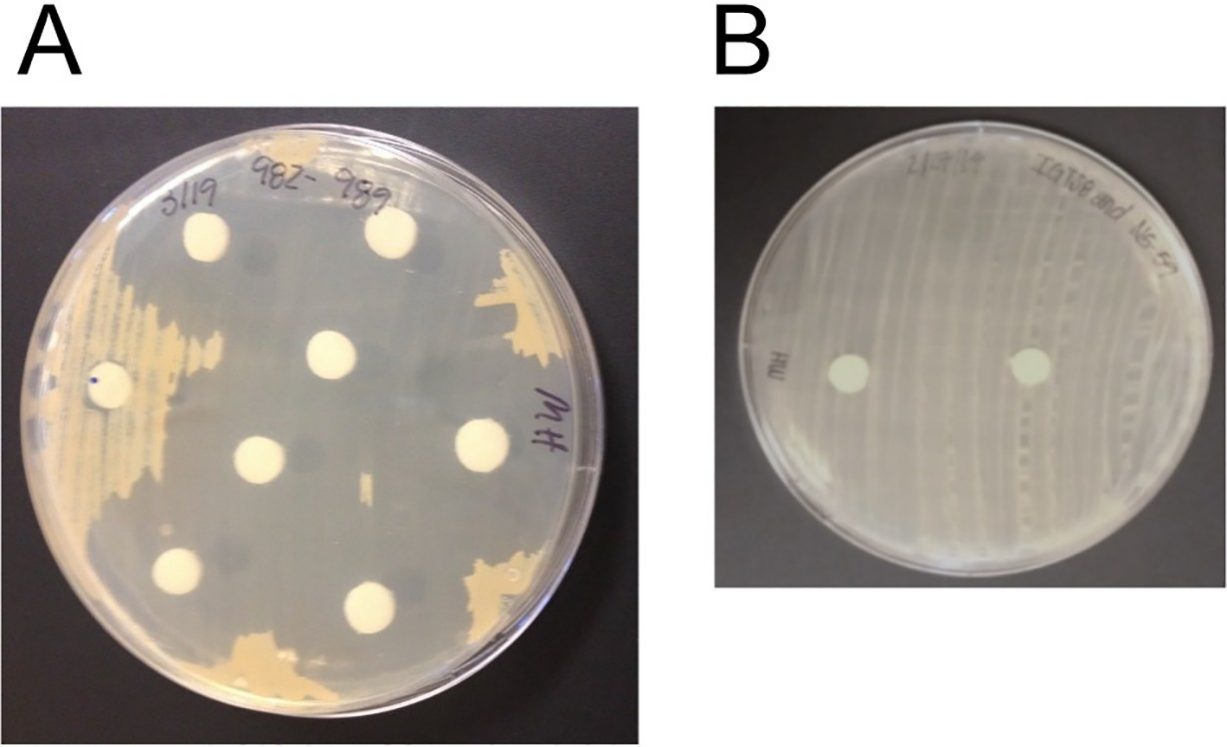
The screening assay to detect non-producing mutant strains. A. In this disk diffusion assay, paper disks were soaked with solvent extracts prepared from agar plate cultures from each of 2,306 mutant strains and placed on a lawn of the sensitive indicator bacterium. One of the mutant extracts (paper disk at 9 o’clock) no longer produces the inhibitory molecule. B. This is a disk diffusion assay with two negative controls. The disk on the left contains an agar plate culture extract from *Rhodococcus erythropolis* strain IGTS8, a strain that does not produce any known inhibitory molecule. The disk on the right was soaked in methanol, a solvent used to prepare the agar extracts.

Random transposon insertional mutagenesis was used to generate random mutations of the MTM3W5.2 genome [18]. These random mutations were screened for variants that no longer produce the inhibitory compound with the aim of identifying genes required to synthesize this molecule. This was accomplished by growing each of 2,306 random mutant strains on an RM plate. After two weeks of growth at 19°C, the agar medium from each of the 2,306 plates was extracted with ethyl acetate and the extracts tested for the presence of the inhibitory compound by the disk diffusion assay (Fig 4). Agar plate extracts that showed no zone (or a very small zone) of inhibited growth of the sensitive indicator bacterium (*R*. *erythropolis* strain IGTS8) were scored as non-producer mutants. From this screen of 2,306 mutants, eight non-producing strains were detected.

A DNA (Southern) hybridization experiment was done to confirm that the non-producing mutant strains were due to single transposon insertions in the chromosome. For DNA from mutant strains RMP 2.31 (S1 Fig, lane 8) and strain RMP 77.23 (lane 9) a single restriction fragment strongly hybridized to the probe DNA containing transposon sequences (S1 Fig). This is strong evidence that these non-producing phenotypes are the result of a single transposon insertion into the chromosome. In addition, the single hybridizing restriction fragment in most mutant DNAs is of a different size indicating that the transposon inserted into different locations in the genome of each mutant strain.

**Fig 5:**
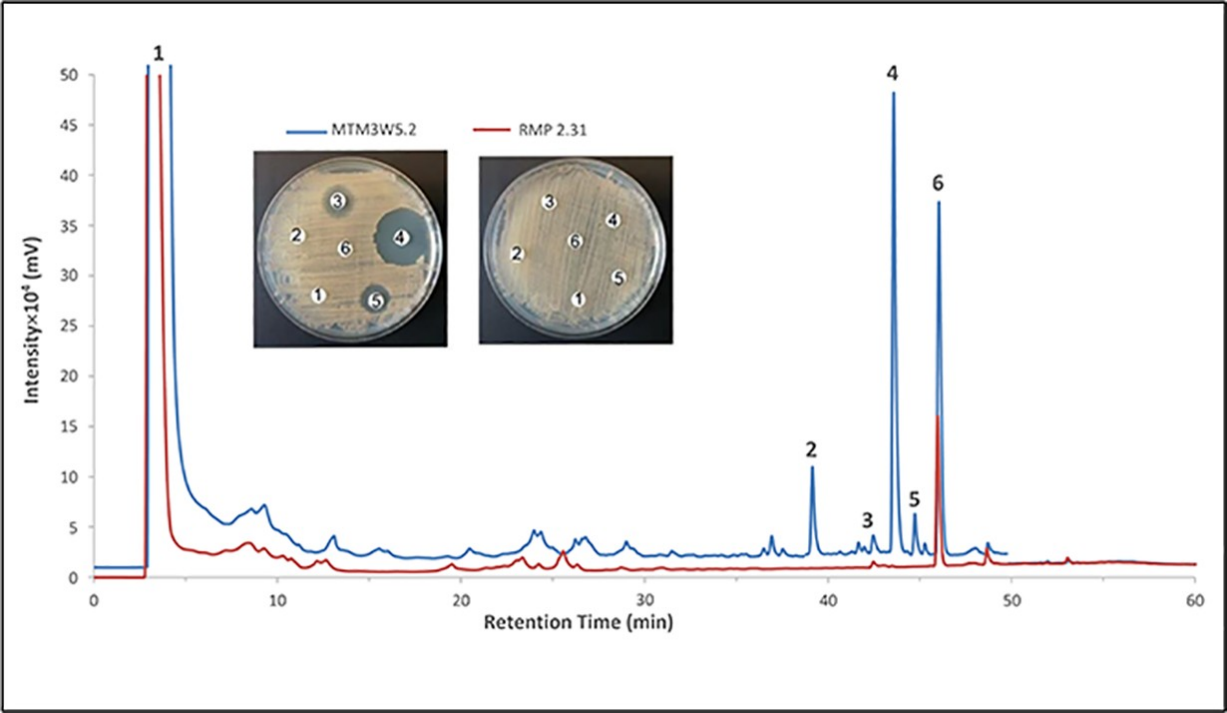
The inhibitory compound is not detected in the culture extract from the mutant strain RMP 2.31. Butanol extracts were prepared from stagnant broth cultures of the parent strain MTM3W5.2 and the non-producing mutant strain RMP 2.31. These extracts were fractionated by HPLC and compared by overlaying the resulting chromatograms. Fractions corresponding to peaks labeled 1 through 6 were also tested for inhibitory activity by the disk diffusion assay (picture inset). Most of the inhibitory activity is present in peak 4 (retention time of 43.5 minutes) from the parental extract (blue line). Peaks 2 through 5 are not detectable in the mutant extract (red line). Peak 6 is present in both culture extracts but does not show any detectable inhibitory activity in the disk diffusion assay (picture inset).

The non-producing phenotype of the mutant strain RMP 2.31 was further established by comparing the bioassay guided HPLC fractionation of broth culture extracts of this strain with the parent strain MTM3W5.2 (Fig 5). Most of the inhibitory activity appears in an HPLC fraction with a retention time of 43.5 minutes and corresponds to a large, well isolated peak (Fig 5, peak number 4). This large peak is completely absent from the mutant culture extract treated in the same way (Fig 5, red line). Peak number 6 (Fig 5) with a retention time of 47 minutes is present in both wild type and mutant culture extracts but does not show any inhibitory activity (Fig 5, picture inset).

### The transposon insertion sites

In the pTNR transposon system [18] the segment of transposed DNA contains a kanamycin resistance marker and an *E*. *coli* plasmid origin of replication. This allows for easy, on step cloning of the flanking DNA adjacent to the site in the *Rhodococcus* genome where the transposon has inserted. The flanking DNA was cloned from mutant strains RMP 2.31 and RMP 77.23 because these strains showed a clear single hybridizing restriction fragment in their chromosome (S1 Fig). From these plasmid clones, the DNA sequence at the insertion site was determined and this sequence information was used to identify the gene interrupted by the transposon DNA. For mutant RMP 2.31, the transposon appears to interrupt a gene similar to a PKS gene, specifically the acyl transferase (AT) enzymatic domain of a modular PKS (S2 Fig). For mutant strain RMP 77.23, the DNA flanking the inserted transposon shows significant similarity to a ketoreductase (KR) domain of a modular PKS gene (not shown).

### The genome of strain MTM3W5.2

The biosynthesis of small polyketide molecules often requires several large modular synthase genes that lie adjacent to each other and are organized into a gene cluster on the chromosome. This gene cluster provides all the enzymatic functions of a shared biosynthetic pathway required to produce the small molecule. However, some of these polyketide gene clusters can be very large, exceeding 100,000 base pairs (bp) in size, making cloning difficult [19]. To identify the gene cluster required to synthesize the inhibitory compound, the genome sequence of strain MTM3W5.2 was determined. The genome is composed of a single circular DNA molecule of 5,665,081 bp with a 68.9 percent GC content and 5,848 predicted open reading frames (ORF). The sequence of the 16S ribosomal RNA gene from MTM3W5.2 shows a 99 percent match (1401 out of 1411 bp) with the 16S rRNA from *R*. *maanshanensis* [17], a soil isolate originally obtained from Maanshan Mountain, China, suggesting a close relation to this previously identified species.

### Biosynthetic gene clusters

To detect potential BGCs, the genome sequence of strain MTM3W5.2 was analyzed using the computational search tool antiSMASH [20]. The latest version of antiSMASH predicted over 70 potential BCGs to be present in the genome of this *Rhodococcus* strain (S2 Table). For example, the gene cluster (cluster 60) at positions 4915866 to 4972505 likely is involved in the synthesis of a compound similar to rhodochelin, a previously characterized siderophore molecule produced by *R*. *jostii* RHA1 [21]. Other BGCs in the MTM3W5.2 genome that are also found in many other *Rhodococcus* genomes include the following. The type I PKS gene BTZ20_5696 (cluster 70) is similar to the last “condensase” of mycolic acid biosynthesis. A butyrolactone gene cluster (cluster 65) may produce a gamma-butyrolactone signaling molecule for the regulation of secondary metabolism. A terpene type BGC (cluster 36) maybe required to produce iron-sulfur cluster protein co-factors. A terpene gene cluster (cluster 40) maybe involved in carotenoid biosynthesis. And finally, an ectoine gene cluster (cluster 42) that may produce this molecule as an osmoprotectant in high salt environments [3]. However, the great majority of these putative gene clusters have very low similarity to previously known genes involved in the synthesis of known secondary metabolites and thus the compound they may produce is, basically, unknown.

**Fig 6:**
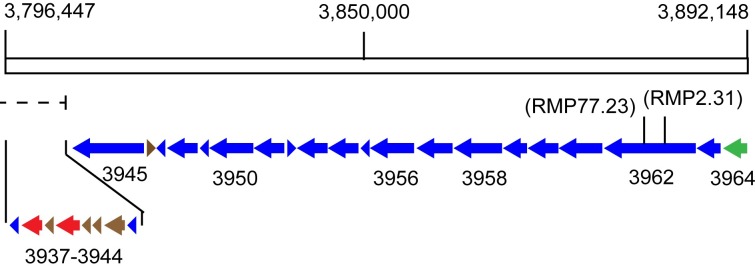
A 98,493 base-pair region of the MTM3W5.2 genome contains the proposed biosynthetic gene cluster required to produce the inhibitory compound. Large arrows and arrow heads indicate proposed genes with numbers designating the specific BTZ20 loci of the genome sequence. The gene cluster likely begins with the regulatory locus BTZ20_3964 (at position 3894940) and may end with the three small loci BTZ20_3939–37 (at position 3796447). This includes a possible NRPS gene BTZ20_3940. The two transposon insertion mutations (RMP 2.31 and RMP 77.23) are shown and map to the same large type I PKS gene BTZ20_3962. These two mutations render a phenotype that no longer produces the inhibitory compound. The dashed line shows the right end of a large (232,272 bp) postulated genome island region that may overlap with the very end of the biosynthetic gene cluster. The open rectangle represents the genomic DNA region containing the gene cluster 46. The color key is: Blue arrows are type I PKS genes, green arrow in a transcriptional regulatory gene, red arrows are NRPS type genes, and brown arrows are other gene classes.

At over 98,000 bp in size, a particularly large gene cluster is found at positions 3796447 to 3894940 (cluster 46) and contains at least 14 different, large type I modular polyketide synthase genes (Fig 6 and Table 2). Based on DNA sequences cloned adjacent to the transposon element pTNR, both the mutant RMP 2.31 and RMP 77.23 appear to have inserted (interrupted) into the largest type I PKS gene, i.e., gene BTZ20_3962 (Fig 6 and Table 2). This very large modular PKS gene contains 3,962 amino acids with 11 enzymatic domains including two substrate determining acyltransferase (AT) domains, one for malonyl-CoA and the other for methylmalonyl-CoA. The site of these two insertional mutations, yielding a non-producing phenotype, is strong evidence that this type I PKS gene and the large gene cluster that it is associated with are required to produce the inhibitory molecule. This gene cluster may also contain a Lux R “solo” type transcriptional regulatory gene (gene BTZ20_3964) that may regulate its expression [22]. Finally, at least two NRPS type genes are predicted to be part of this large gene cluster suggesting that the molecule it produces may contain an amino acid (Table 2, genes BTZ20_3940, and 3938). However, based on the compound’s mass, there is no evidence for this (Fig 3).

**Table 2:** Proposed biosynthetic gene cluster required to synthesize the inhibitory molecule.

Locus/ORF^a^	No. of Amino acids	Proposed function / domains^b^	Similar protein/origin	% Identical /similar amino acids
BTZ20_3964	930	Lux R-type transcriptional regulator	WP_886662186 / *Lentzea kentuckyensis*	35/52
BTZ20_3963	1,054	Type I PKS / AT(MeM)-KS-DH	BAW35616, modular PKS / *Streptomyces* sp. RK95-74	61/75
BTZ20_3962	3,962	Modular type I PKS / KR-DH-AT(Mal)-KS-T- KR-AT(MeM)-KS-T-KR-DH	WP_060953576, type I PKS / *Streptomyces hygroscopicus*	50/62
BTZ20_3961	1,949	Type I PKS / T-KS-AT(MeM)-DH-KR-T	AEP40934, type I PKS / *Nocardiopsis* sp. FU40	53/63
BTZ20_3960	1,529	Type I PKS / T-KR-AT(Mal)-KS	EGX61517, modular PKS / *Streptomyces zinciresistens* K42	54/66
BTZ20_3959	940	Type I PKS / KS-AT(MeM)	AGP60637, hypothetical protein / *Streptomyces rapamycinicus* NRRL 5491	60/71
BTZ20_3958	2,153	Type I PKS / DH-KR-T-KS-AT(MeM)-KR	WP_009715042, PKS / *Streptomyces himastatinicus*	52/65
BTZ20_3957	1,748	Type I PKS / T-KS-AT(Mal)-KR-T	SCF99392, PKS FscD (pimaricinolide synthase) / *Streptomyces* sp. LamerLS-31b	53/65
BTZ20_3956	2,071	Type I PKS / KS-AT(MeM)-KR-T-KS	WP_069865677, PKS / *Streptomyces* sp. SPMA113	55/68
BTZ20_3955	164	Acyltransferase domain protein / -AT(MeM)-	ABB86422, NapD / *Streptomyces hygroscopicus* subsp. duamyceticus	68/77
BTZ20_3954	1,450	Type I PKS / AT(Mem)-KR-T-KS-AT(Mal)	? (BTZ20_3956), PKS / *Rhodococcus* sp. MTM3W5.2	72/81
BTZ20_3953	1,396	Type I PKS / AT(Mal)-DG-KR-T-**TE**	WP_063770247 / *Streptacidiphilus melanogenes*	47/59
BTZ20_3952	162	Beta-ketoacyl synthase domain protein / -KS-	WP_071802865, VOC family protein / *Couchinoplanes caeruleus*	35/51
BTZ20_3951	1,841	Type I PKS / AT(MeM)-T-KS-AT(Mal)-KR	SCL54008, PKS12 / *Micromonospora eburnean*	48/61
BTZ20_3950	2,247	Type I PKS / T-KS-AT(Mal)-DH-ER-KR-T	WP_0830873745, PKS / *Mycobacterium noviomagense*	54/67
BTZ20_3949	551	Beta-Ketoacyl synthase domain protein / -KS-	WP_079133612, PKS / *Streptomyces* sp. FXJ1.172	62/73
BTZ20_3948	1,891	Type I PKS / AT(MeM)-T-KS-AT(Mal)-KR	AGP60636, hypothetical protein / *Streptomyces rapamycinicus* NRRL 5491	52/64
BTZ20_3947	114	Putative chlA1 (phosphopantetheine attachment site domain)	WP_072653505, hypothetical protein / *Streptomyces viridifaciens*	30/48
BTZ20_3946	313	Hypothetical protein (FeII-dependent oxygenase superfamily protein)	WP_0179117885, hypothetical protein / *Burkholderia gladioli*	36/55
BTZ20_3945	3,311	Type I PKS / KS-AT(MeM)-KR-T-KS-AT(Mal)-DH-KR-T	WP_062013672, PKS / *Streptomyces sporocinereus*	51/63
*BTZ20_3944*	210	Alpha/Beta hydrolase family protein (thioesterase domain) / -**TE**-	WP_063920616, thioesterase / *Nocardia soli*	55/69
*BTZ20_3943*	429	CcrA (Crotonyl-CoA reductase) / *ccrA* gene	WP_082844406, crotonyl-CoA carboxylase/reductase / *Nocardia soli*	80/90
*BTZ20_3942*	281	Demethyl-rebeccamycin-D-glucose D-methyl transferase (*rebM* gene)	WP_056603428, SAM-dependent methyltransferase / *Aeromicrobium* sp.	51/63
*BTZ20_3941*	132	Hypothetical protein	WP_021592450, cold-shock protein / *Actinomadura madurae*	32/45
*BTZ20_3940*	548	NRPS/AMP-binding enzyme family / -A-	WP_082844400, long-chain fatty acid CoA ligase / *Nocardia soli*	67/78
*BTZ20_3939*	85	Hypothetical protein	WP_082844398, KR domain-containing protein / *Nocardia soli*	81/116
*BTZ20_3938*	448	NRPS-like protein / -A-	WP_082844399, long-chain fatty acid-CoA ligase / *Nocardia soli*	231/448
*BTZ20_3937*	201	PKS,NRPS-like protein / -KR-	WP_082844398, KR domain-containing protein / *Nocardia soli*	92/112

^a^, Loci in italics are part of a genome island.

^b^, PKS, polyketide synthase; NRPS, non-ribosomal peptide synthetase; Domains: AT, acyltransferase(substrates: Mal, malonyl-CoA; MeM, methylmalonyl-CoA); T, acyl carrier protein; KS, beta-keto synthase; KR, ketoreductase; DH, dehydratase; ER, enoylreductase; TE, thioesterase; A, adenylation (for NRPS)

### Genome island

The genome sequence of strain MTM3W5.2 was analyzed using the Zisland explorer algorithm [23]. This bioinformatics tool can be used to detect potential genome islands based primarily of GC composition bias within the DNA sequence of the island compared to the rest of the genome. One genome island was detected in the genome sequence using this algorithm and is found between positions 3,571,646 and 3,803,918 (not shown). This is a large region of the genome spanning 232,272 base pairs (bp) and contains over 200 genes. Interestingly, the last 4,775 bp of this genome island overlaps with part of the biosynthetic gene cluster (cluster 46, S2 Table) predicted to be required for the synthesis of the inhibitory compound (Fig 6, dashed line). Specifically, the genes BTZ20_3937 to BTZ20_3944 at the end of the gene cluster lie within the right end of the postulated genome island.

## Discussion

A newly isolated *Rhodococcus* strain MTM3W5.2 releases a small inhibitory compound into stagnant culture broths or into the agar of plate cultures. This molecule has a molecular mass of 911.5460 (Fig 3) and a rather narrow spectrum of activity, only inhibiting the growth of other species of *Rhodococcus* and some closely related genera (Table 1). This is somewhat similar to bacteriocins. However, bacteriocins like the lantibiotics and various lipopeptides produced by Gram positive bacteria, are synthesized by the ribosome and usually have a molecular mass greater than 2000 [24].

The inhibitory compound described here is likely to be different from antibiotic type molecules previously reported from *Rhodococcus*. For example, the rhodopeptins are small cyclic peptides that only have activity against fungal organisms [11]. The lariatins are large lasso type peptides greater than 2000 molecular weight [12]. Even the aurachins [13, 14], although small in size (364–395 molecular weight), appear to have a broader spectrum of activity by inhibiting members of Gram positive genera like *Bacillus* and *Arthrobacter* that are not sensitive to the compound described here (Table 1).

Based on the analysis of two non-producing (transposon) mutant strains, a large type I, modular PKS gene appears to be required to synthesize this inhibitory compound. This large PKS gene (BTZ20_3962) is part of a very large BGC containing at least 14 different PKS genes (Table 2, and Fig 6). The “KnownClusterBlast” module of antiSMASH also compared this gene cluster from *Rhodococcus* with known biosynthetic gene clusters that produce previously studied secondary metabolites. In most cases only a minority (less than 50%) of genes in the *Rhodococcus* gene cluster share similar genes with known biosynthetic gene clusters. These include gene clusters for Filipin, a pentaene macrolide with antifungal activity (46% similar)[25]; Nystatin-like compound from *Pseudonocardia* (46%)[26]; and Oligomycin, a macrolide with anticancer and other activities (53%)[27]. Whether the product of this *Rhodococcus* gene cluster is of a similar class to these macrolides is yet to be determined. However, what is clear is that most of the PKS genes found in this gene cluster are more similar to genes found in *Streptomyces* than in known *Rhodococcus* genomes (Table 2 and ClusterBlast results, not shown). This suggests a possible gene exchange origin for this gene cluster. The Zisland Explorer algorithm [23] predicted the presence of a single genome island in the genome of strain MTM3W5.2 that stretches over a region of 232,272 bp. Interestingly, the last 4,775 bp of this genome island overlaps with part of the biosynthetic gene cluster (cluster 46) predicted to be involved in the synthesis of the inhibitory compound (Fig 6, dashed line). Specifically, the genes BTZ20_3937 to BTZ20_3944 at the end of the gene cluster lie within the right end of the postulated genome island. These genes include a NRPS with a solitary adenylation domain, a potential methytrasferase, and a protein with a solitary thioesterase domain (Table 2), all possibly involved in the addition of an amino acid to the core structure of the inhibitory compound. Although there is no evidence for an amino acid, perhaps by chance, the acquisition of this genome island fused these NRPS domains to this predominately polyketide biosynthetic pathway creating a new variant form of the inhibitory compound. Such a scenario is not without precedent in *Rhodococcus*. Kurosawa and co-workers reported the ability of *Rhodococcus fascians* to produce a novel variant form of streptomycin following gene exchange in a co-culture with *Streptomyces padanus* [28]. However, based on the precise mass of this molecule (Fig 3), no nitrogen atoms are predicted to be present in this compound.

Finally, at or near the beginning of the proposed biosynthetic gene cluster is a gene (BTZ20_3964) that is similar to a Lux R-type transcriptional regulatory gene (Table 2). Normally, Lux R regulators are part of a Lux I/Lux R type quorum sensing system in which small chemical signals like acyl-homoserine lactones (produced by the Lux I synthase) regulate phenotypes like bioluminescence in a cell density manner [29]. The gene BTZ20_3964, however, appears to be a Lux R “solo”: A Lux R-type transcriptional regulatory protein not associated with a matching Lux I synthase [22]. Such Lux R “solos” have been previously described as positive regulators of antibiotic biosynthesis in *Streptomyces* [25, 30] and could be the regulator for this *Rhodococcus* gene cluster as well.

The ability to exploit this *Rhodococcus* compound for therapeutic purposes against *R*. *equi* infections will require a detailed determination of its chemical structure. Even so, it may be possible to develop the producer strain MTM3W5.2 into a biocontrol agent against *R*. *equi* [31]. *R*. *equi* appears to reside in manure laden farm soils and infection of foals occurs primarily by the inhalation of airborne dust containing the pathogen [7]. It may be possible to reduce the level of contamination of barn yard soils with *R*. *equi* by using strain MTM3W5.2 as a potential biocontrol agent.

## Materials and methods

### Bacterial strains and growth media

*Rhodococcus* strains newly isolated and identified for this study are listed in S1 Table. Methods used to culture and identify this collection of strains are described in the supplemental material. RM (rich medium, supplemental material, S1 File) was used to culture most strains of *Rhodococcus* at either 20°C or 28°C. Mueller-Hinton medium (Difco) was used for the disk diffusion assays.

### Culture extracts

Extracts were prepared from agar plate cultures [32] and used to screen our *Rhodococcus* collection for antibacterial compounds and to screen transposon mutants for variants that no longer produce the inhibitory molecule. Each *Rhodococcus* strain was inoculated for confluent growth on an RM plate. After incubation at 21°C for two to three weeks, cells were washed off the surface and the agar plate was cut up into 1 cm square cubes and placed into a 250 ml beaker. The agar squares were twice soaked in 50 ml of ethyl acetate and the organic extract was decanted, pooled (100 ml), and allowed to dry. The dried extract was dissolved in 1 ml of methanol and this was used to test for antibacterial activity in a disk diffusion assay. For the disk diffusion assay, paper disks (7 mm in diameter) made from thick Whatman blotting paper, were soaked in 50 ul of each culture extract, allowed to dry, and then placed on a Muller-Hinton agar plate inoculated with a sensitive indicator bacterium. One of three indicator bacteria were used in each assay: *Micrococcus luteus*, *Escherichia coli*, and *R*. *erythropolis* IGTS8. The solvent control was a disk soaked in methanol.

Culture extracts were also prepared from stagnant RM broth cultures of the producer strain MTM3W5.2. Under stagnant conditions, MTM3W5.2 grows as a biofilm on the surface of the broth. After removal of cells by centrifugation, the culture broth was extracted with 1-butanol. The organic phase was recovered via a separatory funnel, dried, and dissolved in isopropanol.

### Preparation of electro-competent *Rhodococcus* cells

*Rhodococcus* strain MTM3W5.2 was grown at 28°C with shaking in 50 mls of RM broth to an OD_600_ of between 2 and 4. Cells were harvested by centrifugation and then re-suspended in 30 ml of ice cold, sterile, 10% (v/v) glycerol. The cells were incubated on ice for 10 minutes and then harvested again by centrifugation. The cell pellet was again re-suspended in just 15 ml cold, sterile, 10% glycerol and left on ice for 10 minutes. Finally, the cells were harvested by centrifugation and the cell pellet was re-suspended for a final time in just 0.6 ml of sterile 10% glycerol. Aliquots (120ul) of this final cell suspension were stored at -80°C.

### Transposon mutagenesis

Upon transformation of the pTNR plasmid into *Rhodococcus* cells, the transposable IS1415 DNA moves from the plasmid to a random site in the *Rhodococcus* genome [18]. The mobilized DNA carries a kanamycin resistance gene and this allows selection for this event, while the plasmid is unable to replicate in the *Rhodococcus* host and is presumably lost. For electroporation of the pTNR plasmid into the parent strain MTM3W5.2, 100–300 ng (2ul) of the plasmid DNA was mixed with 100 ul of electro-competent MTM3W5.2 cells previously thawed on ice. In an electroporation cuvette, cells plus DNA were pulsed at 2,500 volts. After electroporation, the cells were incubated in RM broth at 28°C for 3 hours. Then dilutions were plated on RM plates containing 200 ug/ml kanamycin. Transformant colonies appeared after incubation at 28°C in about 5 to 6 days.

### Isolation of total DNA from *Rhodococcus* cells

An over night seed culture (0.5 ml of a 2 ml LB broth incubated at 28°C) of MTM3W5.2 was used to inoculate 25 ml of LB broth in a 250 ml flask. After shaking for 20 hours at 28°C, ampicillin was added to the culture broth to a final concentration of 0.4 mg/ml. After an additional 20 hours of incubation (shaking at 28°C), the culture cells were harvested by centrifugation and the cell pellet was washed in 3 ml of TE buffer (10 mM Tris-HCl, 1 mM EDTA, pH 8.0). The cell wash was again collected by centrifugation and the final cell pellet was re-suspended in 1 ml of TES buffer (50 mM Tris-HCl, 1 mM EDTA, 25% sucrose, pH 8.0). To this was added 1 ml of TE buffer containing 50 mg of freshly prepared lysozyme. This cell suspension was then incubated with periodic agitation for a minimum of 4 hours at 37°C. Then 30 ul of 20% SDS plus 10 ul of RNase (33ug/ml final concentration) was added to 0.5 ml of the cell suspension in a 1.5 ml microfuge tube. After mixing, the cell lysate was incubated at 55°C for 15 minutes. Proteinase K (30 ug) was then added and the lysate was incubated at 55°C for an additional 15 minutes. The cell lysate was then extracted with an equal volume (0.5 ml) of phenol (saturated with TE, pH 8.0), followed by extraction of the top aqueous phase with phenol plus chloroform-isoamyl alcohol (24:1). Finally, the aqueous phase was extracted twice with just chloroform-isoamyl alcohol. After these extractions, 1 ml of cold ethanol (100%) was slowly added to the aqueous phase and the tube was inverted many times to mix. The precipitated chromosome was then spooled onto the end of a pipet tip and placed in 0.4 ml of 70% ethanol. The DNA chromosome was centrifuged followed by removal of all alcohol and briefly air dried. The DNA was re-dissolved in 100 ul of 0.1 TE and stored at 4°C.

### Recovery of transposon insertion sites

The IS1415 DNA that transposes into the chromosome of *Rhodococcus* contains an *E*. *coli* plasmid *ori* site. This origin of replication can be used to quickly clone *Rhodococcus* DNA that flanks each side of the inserted transposon DNA. Total genomic DNA prepared from each mutant strain was digested with the restriction endonuclease *Xho* I. This digested genomic DNA was then self-ligated based on procedures described by Withers et al. [33]. The *Xho* I digested chromosome (2 ng) was added to a 15 ul ligation mix containing T4 DNA ligase and incubated at room temperature for 2 hours. This was followed by heat inactivation at 65°C for 10 minutes. The ligation mix was used directly to transform electro-competent *E*. *coli* DH5alpha cells, followed by incubation on LB plates containing 35 ug per ml of kanamycin. Transformed colonies appeared in about 24 hours.

Plasmids recovered from transformed colonies were used to sequence the *Rhodococcus* DNA that flanks each end of the inserted transposon. This was done using two outwardly directed primers: pTNR199, 5’-TGAGTGCTTGCGGCAGCGTCTAG and pTNR2611, 5’-GATCCTTTGATCTTTTCTACGGGG, that are located at the ends of the transposed DNA. DNA sequencing was determined using a Beckman CEQ 8000 automated DNA sequencer by the East Tennessee State University molecular biology core facility.

### Genome sequencing

Long sequence reads using PacBio sequencing technology was used to determine the complete genome sequence code for strain MTM3W5.2. This resulted in 37.907X genome coverage and sequence reads were assembled using Canu v.1.2 method (Genomics Resource Center, Institute of Genome Sciences, University of Maryland School of Medicine). The complete (and annotated) genome sequence is deposited under GenBank accession number CP019572.1.

### Purification of the inhibitory molecule

Stagnant broth cultures of the parent (MTM3W5.2) or mutant (RMP 2.31) strains were extracted with n-butanol and then dried. The dried extract was dissolved in a small (10 ml) volume of isopropanol and then applied to a Sephadex LH-20 column and eluted with methanol. Column fractions were monitored for absorbance at 210 to 280 nm wave lengths and for inhibitory activity using the disk diffusion assay. Fractions showing inhibitory activity were pooled, dried, and re-dissolved in isopropanol. High performance liquid chromatography (HPLC) of the crude fractions was carried out on a Shimadzu LC-1OAS instrument equipped with a SCL-1OAVP system controller and a SPD-1OA UV-Vis detector. Primary purification of the pooled active fractions was performed on a semi-preparative, Hamilton 12–20 um, PRP-1 polymeric, reverse phase, 100 Å column (250 × 22.2 mm) (Hamilton, Reno, NV, USA). This was followed by repetitive purification on an analytical Kinetex, 5um EVO C18, 100 Å column (250 mm × 4.6 mm) (Phenomenex, Torrance, CA, USA), utilizing a mixture of water (solvent A) and acetonitrile + 0.05% formic acid (solvent B) as mobile phase. The gradient elution was achieved with a linear increase of solvent B (30 to 100%) within 45 minutes, followed by holding in 100% B for an additional 15 minutes at 254 nm wave length for detection and a flow rate of 1.8 ml per minute.

### HPLC analysis of the crude extract

Initially the HPLC system was programmed on a Kinetex, 5um phenyl-hexyl column, 100 Å column (250 mm × 4.6 mm) (Phenomenex, Torrance, CA USA) to analyze the n-butanol culture extracts of the parent strain MTM3W5.2 and the non-producing mutant strain RMP 2.31 (Fig 5). A linear gradient elution method was used by increasing the methanol concentration from 40% to 100% over a 45 minute period, then holding the solvent B at 100% for an extra 15 minutes. The phenyl-hexyl column was stabilized for 30 minutes using a methanol–water mixture (40:60) prior to injecting the sample. The injection volume of crude extract was 20 ul.

### Mass spectroscopy (HRESI-MS)

The high resolution, accurate mass spectra were acquired on a Bruker maXis II Quadrupole Time-of-Flight (Q-TOF) high-resolution mass spectrometer (Bruker Daltonics, Bremen, Germany). The measurements were performed in an electrospray ionization (ESI) positive ion mode, scanning mass range from *m/z* 50 to *m/z* 3000. The pure compound sample (peak 2, Fig 2) was dissolved in acetonitrile-water (50:50) plus 0.1% formic acid and placed on the auto-sampler for injection.

## Supporting information

S1 File Supplemental methods. (DOCX)

S1 Fig Southern blot analysis of pTNR transposon insertions. (DOCX)

S2 Fig Amino acid sequence alignment. (DOCX)

S1 Table *Rhodococcus* strains isolated and identified for this study. (DOCX)

S2 Table Potential biosynthetic gene clusters in the genome of strain MTM3W5.1. (DOCX)
