# Supplementary material for: An inhibitory compound produced by a soil isolate of Rhodococcus has strong activity against the veterinary pathogen R. equi
Source: PLoS One. 2018 Dec 28;13(12):e0209275. doi: 10.1371/journal.pone.0209275 (PMC6310278; doi:10.1371/journal.pone.0209275)
Supplement: S1 File — (DOCX) [file pone.0209275.s001.docx]

Supplementary Online Material

Ward AL, et al. 2018. An inhibitory compound produced by a soil isolate of *Rhodococcus* has strong activity against the veterinary pathogen *R. equi*.

Materials and Methods

**Collection of soil samples**. Many different types of soil samples from different geographic locations were collected to maximize the diversity of new *Rhodococcus* strains recovered from these environmental sources [S1]. Most samples were surface soils about 5-20 cm deep. Soil samples included arid soils, lake and river sediments, mountain and forest soils, and soils likely to be contaminated with petrochemicals (S1 Table). After collection, soil samples were sealed in plasitic bags or other sealed containers and stored under refrigeration until culturing.

No specific permissions were required to obtain these soil samples. These soil samples were provided by authors of this study, their students, or by park officials. Soil samples were from surface locations, were very small (generally less than 5 grams), and had no environmental impact on any endangered of protected species.

**Growth media**. Rich medium (RM) contains glucose (10g/liter), nutrient broth (8g/liter), and yeast extract (0.5g/liter) in distilled water. Most strains of *Rhodococcus* were cultured in RM at either 21^o^C or 28^o^C. The defined medium M3 was as described by Rowbotham and Cross [S2] and was used for the heat shock method of enrichment culturing. Defined basal medium (DE) was as described [S3]. DE supplemented with 0.5% acetonitrile was used for the acetonitrile enrichment culture method. Bushnell-Haas (BH) medium was used for the hexadecane enrichment culturing method. BH is composed of KH_2_PO_4_ (2g/liter), K_2_HPO_4_ (2g/liter), NH_4_NO_3_ (2g/liter), MgSO_4_*7H_2_O (0.4g/liter), FeCl_3_ (0.1g/liter), CaCl_2_*2H_2_O (0.04g/liter) in distilled water (heat to dissolve, then autoclave).

**Enrichment culturing to recover *Rhodococcus***. Three different enrichment culturing methods were used to increase the percentage of *Rhodococcus* strains recovered from complex soil communities. The first method utilizes a rigorous heat shock of the soil sample followed by plating on the defined growth medium M3 supplemented with the antifungal agent cyclohexamide [S2]. An initial soil slurry (1g of soil in 10 ml of water) was diluted ten fold and then placed in a 55^o^C water bath for 6 minutes. The heat treated soil slurry was cooled and then 100 μl was spread onto the surface of M3 plates and incubated at room temperature (21^o^C) for 1 to 2 weeks. Individual colonies were re-streaked on RM plates to enhance expression of pigments commonly seen in *Rhodococcus*. Although many genera of bacteria can survive this heat treatment, nonetheless, a high percentage (about half) of new *Rhodococcus* strains were recovered using this culturing method (Table S1).

A second enrichment method used to isolate new strains was a culturing scheme using hexadecane as the sole source of carbon for growth. Rhodococci are well known for their ability to degrade a variety of chemical pollutants from simple hydrocarbons to chlorinated polycyclic aromatics and have been recovered from oil contaminated environments [S4]. Hexadecane was added to 10 ml of BH broth (in a 50 ml flask) to a final concentration of 0.1%. One gram of soil sample was dispersed into this broth and allowed to incubate (shaking) at 28^o^C for 7 days. After 7 days the soil was allowed to settle and 1 ml of this culture was transferred to 10 ml of fresh BH broth containing hexadecane and again incubated with shaking for 7 days. This process of sub-culturing was repeated for a total of 4 subcultures. At this point 100 μl of the culture was spread onto the surface of a BH agar plate. For growth on BH agar plates, hexadecane was provided by soaking a 2 cm square piece of Whatman 3MM chromatography paper that was then placed on the lid of an inverted glass petri dish containing the inoculated BH agar. After sealing the plates, the bacteria can use the volatile phase of the compound as a source of carbon for growth. About 20 new *Rhodococcus* strains were isolated from both clean soils and soil samples likely to be contaminated with petrochemicals using this method.

Finally, a third enrichment culturing method used acetonitrile as a sole source of both carbon and nitrogen for growth due to the presence of a nitrile hydrolase that can degrade nitrile compounds [S5]. Several studies have reported the use of acetonitrile, as well as, other nitrile compounds in enrichment culturing to recover nitrile hydrolyzing bacteria from the environment [S6]. DE broth containing acetonitrile (0.5% final concentration) was inoculated with one gram of sample. This soil culture was subjected to the same four sequential sub-culturing process as described for hexadecane. This enrichment culturing process was done in broths or on plates. After the last subculture, individual colonies were re-streaked on RM plates to observe pigmentation of colonies. Twenty-two new strains of *Rhodococcus* were isolated from mostly pristine soil samples using acetonitrile enrichment culturing (Table S1).

**Identification of *Rhodococcus* strains**. Newly purified strains were identified as *Rhodococcus* based on Gram stain appearance (Gram positive rods and/or cocci), colony pigmentation (cream, yellow, orange, or red) and finally the DNA sequence of their 16S rRNA gene. A single pure colony was used as the DNA template to amplify the 16S rRNA gene from each newly recovered soil isolate. The forward primer 63f (5’-CAGGCCTAACACATGCAAGTC) and the reverse primer 1387r (5’-GGGCGGWGTGTACAAGGC, where W is either A or T) were used to amplify the bacterial 16S rRNA gene by PCR [S7]. The same primers were used to generate the DNA sequence of the nearly complete gene from the PCR amplified DNA (DNA sequencing service, Univ. of Tennessee, Knoxville). The DNA sequence of each amplified DNA was compared to the 16S rRNA genes of known bacteria using both the Ribosome Database Project (http//rdp.cme.msu.edu) and the BLAST alignment with GenBank database (NCBI).

References

S1. Fierer N, Jackson RB. 2006. The diversity and biogeography of soil bacterial communities. Proc Natl Acad Sci USA 103:626-631.

S2. Rowbotham TJ, Cross T. 1977. Ecology of *Rhodococcus coprophilus* and associated actinomycetes in fresh water and agricultural habitats. J Gen Microbiol 100:231-240.

S3. Langdahl BR, Bisp P, Ingvorsen K. 1996. Nitrile hydrolysis by *Rhodococcus erythropolis* BL1, an acetonitrile-tolerant strain isolated from a marine sediment. Microbiol 142:145-154.

S4. de Carvalho CCR, da Fonseca MMR. 2005. The remarkable *Rhodococcus erythropolis*. Appl Microbiol Biotechnol 67:715-726.

S5. Yam KC, Okamoto L, Roberts JN, Eltis LD. 2011. Adventures in *Rhodococcus* – from steroids to explosives. Can J Microbiol 57:155-168.

S6. Layh N, Hirrlinger B, Stolz A, Knackmuss H-J. 1997. Enrichment strategies for nitrile-hydrolysing bacteria. App Microbiol Biotechnol 47:668-674.

S7. Marchesi JR, Sato T, Weightman AJ, Martin TA, Fry JC, Hiom S, Wade W. 1998. Design and evaluation of useful bacterium-specific PCR primers that amplify genes coding for bacterial 16S rRNA. Appl Environ Microbiol 64:795-799.

S8. Goodfellow M, Sangal V, Jones AL, Sutcliffe IC. 2015. Charting stormy waters: A commentary on the nomenclature of the equine pathogen variously named *Prescottella equi*, *Rhodococcus equi* and *Rhodococcus* *hoagii*. Equine Veterinary Journal 47:508-509.
