# Supplementary material for: An inhibitory compound produced by a soil isolate of Rhodococcus has strong activity against the veterinary pathogen R. equi
Source: PLoS One. 2018 Dec 28;13(12):e0209275. doi: 10.1371/journal.pone.0209275 (PMC6310278; doi:10.1371/journal.pone.0209275)
Supplement: S1 Fig — (DOCX) [file pone.0209275.s002.docx]

S1 Fig. Southern blot analysis of pTNR transposon insertions. (a) Total DNA prepared 483 from each non-producing mutant strain was digested with XhoI and the resulting restriction fragments were separated by agarose gel electrophoresis. Lane 2 contains digested DNA from the non-producing mutant strain RMP 71.7. Lane 3 is mutant RMP 71.3, lane 4 is RMP 70.47, lane 5 is RMP 82.5, lane 6 is RMP 46.37, lane 7 is RMP 46.37, lane 8 is RMP 2.31, and lane 9 is RMP 77.23. Lane S contains lambda phage DNA digested with HindIII as a molecular size standard. Lanes 1 and 10 contain no DNA. Lane 11 contains the pTNR plasmid as a positive control DNA. (b) A Southern blot of the agarose gel in (a) was hybridized to biotin labeled pTNR plasmid DNA serving as the probe. Except for mutant strain RMP 71.7 (lane 2), DNAs from all the non-producing mutant strains show at least one restriction fragment that hybridizes to the probe indicating the presence of transposon DNA. The location of λ DNA-HindIII restriction fragments is shown on the left in kilobase pairs. (c) Total DNA from the parental strain MTM3W5.2, serving as a negative control, was digested with SacI and the resulting fragments were separated by agarose gel electrophoresis (lane 1). Lane S contains λ DNA digested with Hind III. Lane 2 contains pTNR plasmid DNA. (d) A Southern blot of the agarose gel in (c) was hybridized to biotin labeled pTNR DNA as a probe. Note that the probe only hybridizes to itself (lane 2) but does not hybridize to DNA from the producer strain MTM3W5.2 (lane 1).
