# Supplementary material for: An inhibitory compound produced by a soil isolate of Rhodococcus has strong activity against the veterinary pathogen R. equi
Source: PLoS One. 2018 Dec 28;13(12):e0209275. doi: 10.1371/journal.pone.0209275 (PMC6310278; doi:10.1371/journal.pone.0209275)
Supplement: S2 Fig — (DOCX) [file pone.0209275.s003.docx]

S2 Fig. The transposon insertion site in mutant strain RMP 2.31 interrupts a gene similar to a PKS gene from *Streptomyces*. The “Query” amino acid sequence was translated from the DNA sequence flanking the transposon insertion from mutant strain RMP 2.31. A BLAST sequence alignment analysis with the GenBank database indicates that this *Rhodococcus* sequence is significantly similar to part of a beta-ketoacyl synthase encoded by a modular PKS gene from *Streptomyces* sp. PVA 94-07. The subject (sbjct) amino acid sequence is from the GenBank protein, sequence ID: ref-WP_023415574. The boxed sequence labeled [KS] is similar to the ketosynthase region of a PKS. The boxed sequence labeled [AT] is similar to the acyl transferase region of a PKS.
