# Supplementary material for: An inhibitory compound produced by a soil isolate of Rhodococcus has strong activity against the veterinary pathogen R. equi
Source: PLoS One. 2018 Dec 28;13(12):e0209275. doi: 10.1371/journal.pone.0209275 (PMC6310278; doi:10.1371/journal.pone.0209275)
Supplement: S1 Table — (DOCX) [file pone.0209275.s004.docx]

S1 Table. *Rhodococcus* strains isolated and identified for this study.

| Strain | Soil location /geographic coordinates | Enrichment method | Extract screened |
| --- | --- | --- | --- |
| A2M3R1 | Mali, Africa / 12.667538,-8.196504 | Heat (M3) | Agar, Broth |
| A2M3C2 | Mali, Africa / “ | Heat (M3) | Agar, Broth |
| A2M3O7 | Mali, Africa / “ | Heat (M3) | Agar, Broth |
| A2M3O6 | Mali, Africa / “ | Heat (M3) | Agar, Broth |
| ACM3C1 | Cliffside, Alaska / 61.194436,-149.711706 | Heat (M3) | Agar, Broth |
| ACM3C8 | Cliffside, Alaska / “ | Heat (M3) | Agar, Broth |
| ACM3O13 | Alaska / “ | Heat (M3) | Agar, Broth |
| ACM3W1 | Alaska / “ | Heat (M3) | Agar, Broth |
| ACM3W5 | Alaska / “ | Heat (M3) | Agar, Broth |
| ACM3W7 | Alaska / “ | Heat (M3) | Agar, Broth |
| ACM3W17D | Alaska / “ | Heat (M3) | Broth |
| ACM3W20 | Alaska / “ | Heat (M3) | Agar, Broth |
| AHACP1 | Roan Mt. TN /36.105199,-82114645 | Acetonitrile | Agar |
| AHACY1 | Roan Mt. TN / “ | Acetonitrile | Agar |
| AHHSO12 | Roan Mt. TN / “ | Heat (M3) | Agar |
| AJACP1 | Roan Mt. TN / “ | Acetonitrile | Agar |
| AJHSO1 | Roan Mt. TN / “ | Heat (M3) | Agar |
| ASM3W2 | Aruba / 12.498556,-69.947037 | Heat (M3) | Agar, Broth |
| BEM3C1 | Kingsport, TN / 36.547002,-82.558976 | Heat (M3) | Broth |
| BIM3W4 | Butler, TN / 36.354571,-82.032007 | Heat (M3) | Broth |
| BMHXW5 | Kingsport, TN / 36.547002,-82.558976 | Hexadecane | Broth |
| BMHXM5 | Kingsport, TN / “ | Hexadecane | Agar, Broth |
| BTHXC2 | Butler, TN / 36.354571,-82.032007 | Hexadecane | Agar, Broth |
| BTHXC6 | Butler, TN / “ | Hexadecane | Agar, Broth |
| BTHXC10 | Butler, TN / “ | Hexadecane | Agar, Broth |
| CBDET1 | Morristown, TN / 36.214945,-83.294817 | Acetonitrile | Agar |
| CBM3M1a | Morristown, TN / “ | Heat (M3) | Agar |
| CBM3T2 | Morristown, TN / “ | Heat (M3) | Agar |
| CBM3T2 | Morristown, TN / “ | Heat (M3) | Agar, Broth |
| CBM3T7 | Morristown, TN / “ | Heat (M3) | Broth |
| CBM3T10 | Morristown, TN / “ | Heat (M3) | Broth |
| CBM3W9 | Morristown, TN / “ | Heat (M3) | Agar, Broth |
| CBM3W11.2 | Morristown, TN / “ | Heat (M3) | Agar, Broth |
| CODEO8 | Cosby, TN / 35.818603,-83.247504 | Acetonitrile | Agar, Broth |
| CODET7 | Cosby, TN / “ | Acetonitrile | Agar, Broth |
| CODET15 | Cosby, TN / “ | Acetonitrile | Agar, Broth |
| CODEW2 | Cosby, TN / “ | Acetonitrile | Agar, Broth |
| CODEY4 | Cosby, TN / “ | Acetonitrile | Broth |
| DCBHXW1 | Tims Ford, TN / 35.225377,-86.273723 | Hexadecane | Agar |
| DSM3C1 | Kingsport, TN / 36.547002,-82.558976 | Heat (M3) | Agar, Broth |
| DSM3C2 | Kingsport, TN / “ | Heat (M3) | Broth |
| EMHXO1 | Embreeville, TN / 36.182880,-82.459033 | Hexadecane | Agar, Broth |
| EMHXC1 | Embreeville, TN / “ | Hexadecane | Agar, Broth |
| ERBAHXW1 | Elk River, TN / 36.312924,-81.991508 | Hexadecane | Agar |
| ERBAHXO1 | Elk River, TN / “ | Hexadecane | Agar |

S1 Table (cont.)

| Strain | Soil location / geographic coordinates | Enrichment method | Extract screened |
| --- | --- | --- | --- |
| EZDET1 | Morristown, TN / 36.214945,-83.294817 | Acetonitrile | Agar |
| KCHXC3* | Kingsport, TN / 36.547002,-82.558976 | Hexadecane | Agar*, Broth |
| KCHXW3 | Kingsport, TN / “ | Hexadecane | Agar, Broth |
| MSYHXW2 | Masaya, Nicaragua / 11.988273,-86.156235 | Hexadecane | Agar, Broth |
| MTDEO2 | Morristown, TN / 36.214945,-83.294817 | Acetonitrile | Agar, Broth |
| MTDEO9 | Morristown, TN / “ | Acetonitrile | Agar, Broth |
| MTDEY3 | Morristown, TN / “ | Acetonitrile | Agar, Broth |
| MTM3O9 | Morristown, TN / “ | Heat (M3) | Agar, Broth |
| MTM3O9a | Morristown, TN / “ | Heat (M3) | Agar, Broth |
| MTM3O2 | Morristown, TN / “ | Heat (M3) | Agar, Broth |
| MTM3O6.2 | Morristown, TN / “ | Heat (M3) | Agar, Broth |
| MTM3T5 | Morristown, TN “ | Heat (M3) | Agar, Broth |
| MTM3T10 | Morristown, TN / “ | Heat (M3) | Agar, Broth |
| MTM3W5.2* | Morristown, TN / “ | Heat (M3) | Agar*, Broth |
| MTM3O2 | Morristown, TN / “ | Heat (M3) | Agar, Broth |
| MTM3O10 | Morristown, TN / “ | Heat (M3) | Agar |
| MTM3O15 | Morristown, TN / “ | Heat (M3) | Agar |
| MTMW12.1a | Morristown, TN / “ | Heat (M3) | Agar, Broth |
| NPDEM14 | Morristown, TN / “ | Acetonitrile | Agar, Broth |
| NPDEO11 | Morristown, TN / “ | Acetonitrile | Agar, Broth |
| NPDEO12 | Morristown, TN / “ | Acetonitrile | Agar, Broth |
| NPDEM13 | Morristown, TN / “ | Acetonitrile | Agar, Broth |
| NPM3T8.1 | Morristown, TN / “ | Heat (M3) | Agar |
| NPM3T11 | Morristown, TN / “ | Heat (M3) | Agar, Broth |
| OCHP1 | ETSU, campus / 36.304639,-82.369766 | Hexadecane | Agar, Broth |
| REACP1 | Roan Mt. TN / 36.105199,-82.114645 | Acetonitrile | Agar |
| REHSC11 | Roan Mt. TN / “ | Heat (M3) | Agar |
| SCTEO3 | Sinking Creek, TN / 36.302762,-82.338581 | Trichloroethylene | Agar, Broth |
| SCM3O11 | Sinking Creek, TN / “ | Heat (M3) | Agar, Broth |
| SIBHXW1 | Hamilton County, TN / 35.194446,-85.17959 | Hexadecane | Agar |
| SIMHXW1 | Hamilton County, TN / “ | Hexadecane | Agar |
| SIMHXW2 | Hamilton County, TN / “ | Hexadecane | Agar, |
| SITVAHXW1 | Hamilton County, TN / “ | Hexadecane | Agar, Broth |
| WDM3P2 | Winged Dear Park, TN /36.388842,-82.36272 | Heat (M3) | Agar, Broth |
| WDM3P13 | Winged Dear Park, TN / “ | Heat (M3) | Agar, Broth |
| WDM3P18 | Winged Dear Park, TN / “ | Heat (M3) | Broth |
| WDM310A | Winged Dear Park, TN / “ | Heat (M3) | Agar, Broth |
| WDM3O7A | Winged Dear Park, TN / “ | Heat (M3) | Agar, Broth |
| WLDET2 | Watauga Lake, TN / 36.354571,-82.032007 | Acetonitrile | Agar |
| WLDEO9* | Watauga Lake, TN / “ | Acetonitrile | Agar* |
| WLDEW1 | Watauga Lake, TN / “ | Acetonitrile | Agar |
| WFDEO8 | Washington County, TN / 36.293686, | Acetonitrile | Agar |
| WFDEM6 | Washington County, TN / -82.481938 | Acetonitrile | Agar |
| WWHXOP1 | Witchita Wildlife Refuge, OK / 34.761760,-98.643466 | Hexadecane | Agar |

*, indicates extracts that showed antibacterial activity.
