# Supplementary material for: An inhibitory compound produced by a soil isolate of Rhodococcus has strong activity against the veterinary pathogen R. equi
Source: PLoS One. 2018 Dec 28;13(12):e0209275. doi: 10.1371/journal.pone.0209275 (PMC6310278; doi:10.1371/journal.pone.0209275)
Supplement: S2 Table — (DOCX) [file pone.0209275.s005.docx]

S2 Table. Proposed biosynthetic gene clusters in the genome of strain MTM3W5.2.

| Cluster No. | Type^a^ | Location | Most similar known cluster (% similar genes) | | MIBiG ID^b^ |
| --- | --- | --- | --- | --- | --- |
| Cluster 1 | Putative | 50593 | - | - | |
| Cluster 2 | T1pks | 69260 | RK-682 BGC (27%) | BGC0000140 | |
| Cluster 3-13 | Putative | 155921 | - | - | |
| Cluster 14 | Bacteriocin | 779299 | - | - | |
| Cluster 15-19 | Putative | 818233 | - | - | |
| Cluster 20 | NRPS | 1067654 | Phosphonoglycans BGC (5%) | BGC0000806 | |
| Cluster 21 | Putative | 1162020 | Ochronotic pigment BGC (50%) | BGC0000918 | |
| Cluster 22 | Putative | 1223165 | - | - | |
| Cluster 23 | Putative | 1247369 | Rifamycin BGC (15%) | BCG0000136 | |
| Cluster 24 | Putative | 1301241 | - | - | |
| Cluster 25 | NRPS | 1346656 | Telomycin BGC (11%) | BGC0001406 | |
| Cluster 26 | Putative | 1588953 | - | - | |
| Cluster 27 | Fatty acid | 1624712 | Lipstatin BGC (21%) | BGC0000382 | |
| Cluster 28 | Putative | 1696309 | - | - | |
| Cluster 29 | NRPS | 1704418 | Cephamycin C BGC (10%) | BGC0000319 | |
| Cluster 30 | Putative | 1824502 | Pyrrolomycin BGC (18%) | BGC0000132 | |
| Cluster 31 | NRPS | 2226799 | Simocyclinone BGC (7%) | BGC0000270 | |
| Cluster 32 | Fatty acid | 2369453 | - | - | |
| Cluster 33 | Putative | 2400627 | Streptomycin BGC (2%) | BGC0000717 | |
| Cluster 34 | Putative | 2458214 | Enduracidin BGC (8%) | BGC0000341 | |
| Cluster 35 | T1pks | 2480142 | Nocathiacin BGC (6%) | BGC0000609 | |
| Cluster 36 | Terpene | 2666300 | Lymphostin BGC (16%) | BGC0001006 | |
| Cluster 37 | NRPS | 2849558 | Lipopeptide 8D1-1 BGC (6%) | BGC0001370 | |
| Cluster 38-39 | Putative | 3179342 | - | - | |
| Cluster 40 | Terpene | 3311218 | Carotenoid BGC (27%) | BGC0000633 | |
| Cluster 41 | Putative | 3424600 | Chlorizaidine A BGC (7%) | BGC0001172 | |
| Cluster 42 | Ectoine | 3548631 | Ectoine BGC (75%) | BGC0000853 | |
| Cluster 43 | T1pks-Nrps | 3641782 | Apoptolidin BGC (35%) | BGC0000021 | |
| Cluster 44 | Fatty acid | 3707194 | - | - | |
| Cluster 45 | Fatty acid | 3729880 | Bottromycin A2 BGC (6%) | BGC0000469 | |
| *Cluster 46* | T1pks | 3783964 | Fillipin BGC (46%) | BGC0000059 | |
| Cluster 47 | Putative | 3952669 | Spinosad BGC (8%) | BGC0000148 | |
| Cluster 48-54 | Putative | 4607540 | - | - | |
| Cluster 55 | Putative | 4607540 | Thiolutin BGC (8%) | BGC0001193 | |
| Cluster 56-59 | Putative | 4639587 | - | - | |
| Cluster 60 | NRPS | 4915866 | Erythrochelin/Rhodochelin BGC (71%) | BGC0000349 | |
| Cluster 61-63 | Putative | 5002334 | - | - | |
| Cluster 64 | Other | 5150447 | Phosphonoglycans BGC (3%) | BGC0000806 | |
| Cluster 65 | Butyrolacton | 5204131 | - | - | |
| Cluster 66-68 | Putative | 5260813 | - | - | |
| Cluster 69 | Saccharide | 5419526 | Chalcomycin BGC (7%) | BGC0000035 | |
| Cluster 70 | T1pks | 5451390 | Echinomycin BGC (11%) | BGC0000339 | |
| Cluster 71-72 | Putative | 5564385 | - | - | |

^a^, T1pks: Type 1 polyketide synthase; NRPS: non-ribosomal peptide synthetase.

^b^, minimum information about a biosynthetic gene cluster (secondarymetabolites.org).
